# Phenomics for photosynthesis, growth and reflectance in *Arabidopsis thaliana* reveals circadian and long-term fluctuations in heritability

**DOI:** 10.1186/s13007-016-0113-y

**Published:** 2016-02-15

**Authors:** Pádraic J. Flood, Willem Kruijer, Sabine K. Schnabel, Rob van der Schoor, Henk Jalink, Jan F. H. Snel, Jeremy Harbinson, Mark G. M. Aarts

**Affiliations:** Laboratory of Genetics, Wageningen University, Wageningen, The Netherlands; Horticulture and Production Physiology, Wageningen University, Wageningen, The Netherlands; Biometris, Wageningen University and Research Centre, Wageningen, The Netherlands; Greenhouse Horticulture, Wageningen University and Research Centre, Wageningen, The Netherlands; Department of Plant Breeding and Genetics, Max Planck Institute for Plant Breeding Research, Cologne, Germany; PhenoVation BV, Wageningen, The Netherlands; Adviesbureau JFH Snel, Wageningen, The Netherlands

**Keywords:** Photosynthesis, Phenomics, Chlorophyll fluorescence, Heritability, High throughput screening, Natural variation, *Arabidopsis thaliana*

## Abstract

**Background:**

Recent advances in genome sequencing technologies have shifted the research bottleneck in plant sciences from genotyping to phenotyping. This shift has driven the development of phenomics, high-throughput non-invasive phenotyping technologies.

**Results:**

We describe an automated high-throughput phenotyping platform, the Phenovator, capable of screening 1440 Arabidopsis plants multiple times per day for photosynthesis, growth and spectral reflectance at eight wavelengths. Using this unprecedented phenotyping capacity, we have been able to detect significant genetic differences between Arabidopsis accessions for all traits measured, across both temporal and environmental scales. The high frequency of measurement allowed us to observe that heritability was not only trait specific, but for some traits was also time specific.

**Conclusions:**

Such continuous real-time non-destructive phenotyping will allow detailed genetic and physiological investigations of the kinetics of plant homeostasis and development. The success and ultimate outcome of a breeding program will depend greatly on the genetic variance which is sampled. Our observation of temporal fluctuations in trait heritability shows that the moment of measurement can have lasting consequences. Ultimately such phenomic level technologies will provide more dynamic insights into plant physiology, and the necessary data for the omics revolution to reach its full potential.

**Electronic supplementary material:**

The online version of this article (doi:10.1186/s13007-016-0113-y) contains supplementary material, which is available to authorized users.

## Background

Photosynthesis is the primary entry point of energy into the biosphere and as such provides the foundation for life on earth. One prominent class of photosynthetic organisms are plants, which are responsible for the vast majority of the energy and biomass influx in the terrestrial biosphere. They are also the basis of our economy, providing the majority of calories necessary to sustain humanity. It is clear that plant photosynthesis is the keystone for our existence, but we know surprisingly little about the extent and basis of variation in this most fundamental of traits [14]. The overarching reason for our lack of knowledge about intraspecific variation in photosynthesis is our inability to efficiently screen large numbers of plants. This epistemic Rubicon must be overcome for our survival, as photosynthesis is the only major productivity-related trait which has yet to be improved [30]. To facilitate this, high-throughput phenotyping of photosynthesis must be developed.

Obtaining phenotypic data is the most time consuming and labour intensive step of many biological experiments [21]. Despite this, the detail and extent of phenotypic data compares poorly with the increasingly complete genotype data now available [13, 22, 37]. This is not only due to the recent advances in genomics but also due to the complex multidimensional nature of phenotypes [21]. The vast number of phenotypic states that a genotype can occupy can be visualised as its phenotypic space, which is often referred to as its phenome. In practice the phenome is a theoretical entity which can never be fully characterised. This was recognised by Houle et al. [22] leading them to propose that phenomics may be understood as the “acquisition of high dimensional phenotypic data on an organism wide scale”.

The phenotype is the result of the interplay between genetics and developmental, environmental and stochastic influences, where the intensity, frequency, order and interaction of these influences affect the outcome. Traditionally, due to its labour intensive nature, phenotyping was only feasible for a single time point on a subset of the traits which comprise the phenome. To reveal, however, the dynamic and variable nature of the phenome, requires numerous measurements across developmental and environmental gradients [21]. Some phenomic (in the sense of Houle et al.) quality datasets for endophenotypes, i.e. transcriptomics, metabolomics, proteomics, ionomics, lipidomics, and even RNA directly undergoing translation (translatomics) have been produced [24, 25]. But as they rely on destructive measurements they only provide a snapshot of the endophenome at the time of measurement. These omics datasets not only lack dynamic insight but they also fail in another import aspect: they give no information about fluxes or growth. Yet, kinetic phenotypes or functional states, such as growth or photosynthesis, provide the most direct and integrative quantification of plant performance [24]. They represent the combined effect of all other phenotypic levels, so the relevance of, for example, variation in gene expression can be assessed at higher organizational levels.

Both photosynthesis and plant growth are ideal traits to assess the functional relevance of endophenotypic omics datasets. Plant size reflects the integration of metabolic and developmental processes and is a good indicator of long term performance whilst photosynthesis and growth rate reflect more immediate physiological responses [9, 12]. Growth responses are most dynamic at the meristematic level [26] which is not amenable to rapid, frequent measurement, in contrast to plant size and photosynthesis, which together provide an ideal phenotypic window into genotype performance.

To this end we set about developing a high-throughput phenotyping platform which would allow us to continuously phenotype a large number of plants for photosynthesis and growth. This will result in phenomic data, though we recognise that the full characterisation of the phenome, namely all possible phenotypic outcomes, across all levels of organisational, developmental and environmental space, is beyond our current capabilities. Nevertheless this is a valuable step forward and will give high-dimensional phenotypic data which, in accordance with Houle et al. [22] can be considered phenomics.

High intensity screening of a particular trait will allow for temporally detailed estimation of heritability. Broad sense heritability is a measure of how much of the phenotypic variance in a population can be attributed to genetic variation rather than other factors, such as a non-uniform environment [42]. It is often used to assess the potential responsiveness of traits to selection, whether natural or artificial [28, 31]. Whilst it is well recognised that heritability is trait, population and environment specific, its variation with time is less well studied. If heritability shows significant time dependence then this will be of interest to breeders and evolutionary biologists, as the time point at which selection occurs will be crucial in determining the selection response. The ability to measure traits multiple times per day for prolonged periods in order to better understand the time-dependency of heritability was an important factor in the design of the phenotyping system we describe here.

## System development

### Design considerations

While the rationale on designing the phenotyping system is described here, the actual experimental conditions and mathematical approaches used to analyse the phenotype data we collected are described in the “Methods” section.

The overarching goal of the phenotyper system, which we named Phenovator, was accurate quantification of the phenotypic variation, so as to estimate the genetic variation, in natural populations of *Arabidopsis thaliana*, using photosynthesis and growth as phenotypic indicators of plant performance. This required that any noise, whether technical, environmental or otherwise, be minimized so that the genetic signal could be accurately assessed. This is particularly important when dealing with traits like photosynthesis, which are environmentally responsive and exhibit limited phenotypic diversity within a species [14]. Thus the plant growth environment should be well controlled to minimize heterogeneity of the environment and allow high reproducibility. Key environmental variables which have a large effect on plant performance and often elicit a phenotypic response, are light, water, temperature and nutrient availability. To control these inputs the Phenovator was located in a climate-controlled growth chamber and equipped with an automated watering system.

To allow repeated measurements of the same plants, and minimize any measurement effects, the measurements must be non-invasive. We therefore developed an image-based phenotyping platform. A balance also had to be struck between the extensive (range) and intensive (detail) capabilities of the Phenovator. We chose to measure a restricted set of phenotypes that are important indicators for plant performance (photosynthetic activity, size, and colour) and to measure these with a high frequency, opting for intensity of measurement. Our optical measurement system was based on a camera. Other camera-based phenotyping systems have been developed and in many the plants are moved to the phenotyping equipment [2, 23, 39, 40]. This has the advantage that the number of plants that can be screened is only limited by the growing area, but the disadvantage is that the plants are not assayed under growth conditions and that the rate of throughput is decreased. In our system we opted to move the camera to the plants and as the camera can be moved at 6 m s^−1^ (much faster than a plant can be safely moved) we can image plants with a high-frequency, but the total growing area that can be imaged is limited by the camera movement system.

**Fig. 1:**
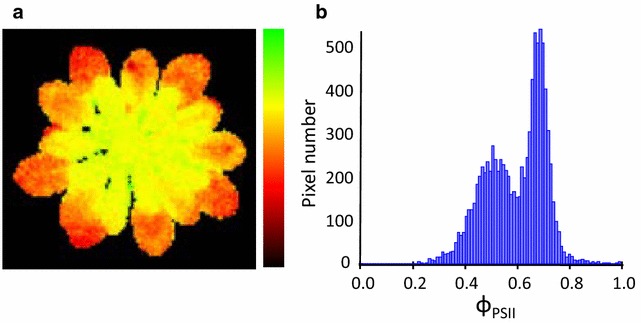
Distribution of photosystem II efficiency (ΦPSII) in a phosphate deficient Arabidopsis. **a** False colour ΦPSII image of a phosphate deficient plant, the *scale bar on the right* shows ΦPSII values from 0 (*black*) to 1 (*green*). **b** Image **a** plotted as a histogram of pixels at specific ΦPSII values. The distribution is bimodal hence the mean value fails to aptly represent the plant’s phenotype

Since many phenotypes show spatial heterogeneity (see Fig. 1 for an example), it was essential to image the entire above-ground part of the plant (roots are outside the scope of this phenotyper). Since our target species, *A. thaliana* (Arabidopsis), forms a rosette, which until flowering is relatively flat, this could be achieved using a single camera. To be able to identify and characterise genetic variation we needed sufficient throughput to screen populations suited for genetic mapping, such as recombinant inbred line (RIL) populations or genome wide association (GWA) panels. The latter populations usually consist of 300 or more genotypes [27] which with four replicates per genotype yields a minimum screening capacity of 1200 plants. To capture short-term changes in the phenotype it was decided that it should be possible to measure all plants within 60 min. Finally it was essential that the entire system was automated, with control and data storage outside the growth room to minimize environmental fluctuations (particularly carbon dioxide) due to people entering the room.

### System design

**Fig. 2:**
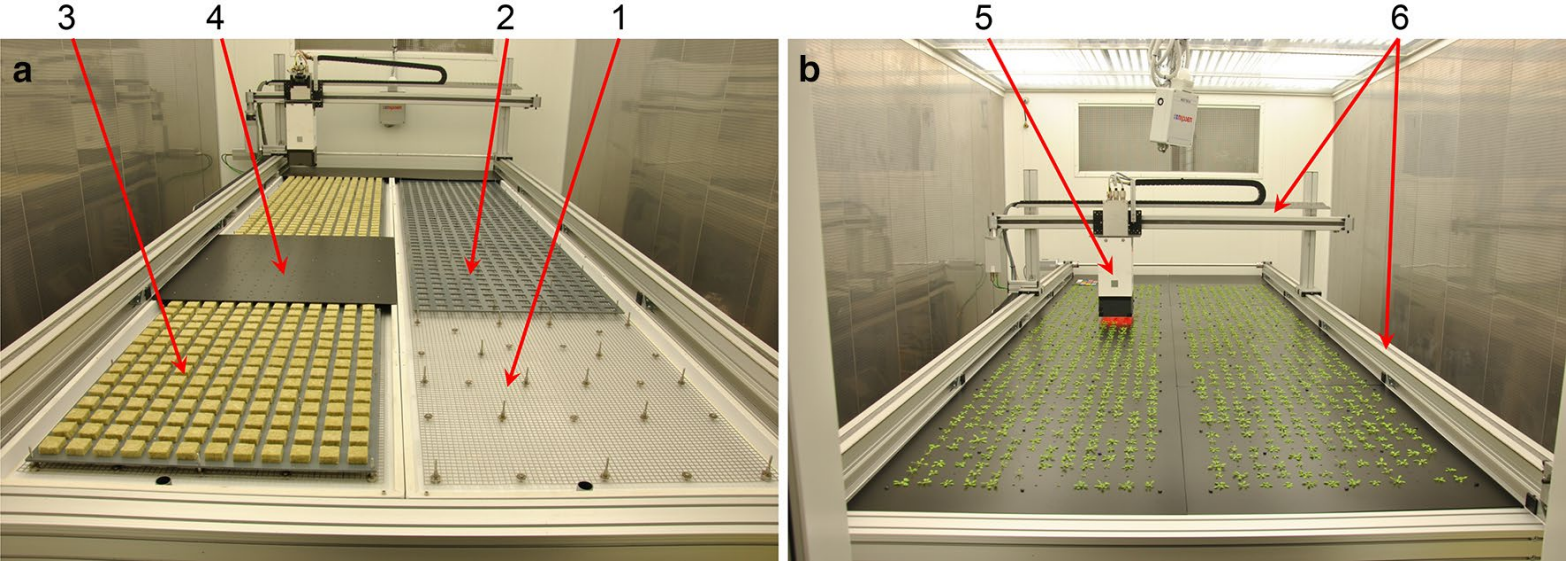
The Phenovator. **a** The set-up of the growth system. *1* Support grid for the rockwool blocks with support pins for the rockwool block spacing plate and the top plate, *2* the rockwool block spacing plate to position and hold the blocks, *3* this grid with rockwool blocks in place, and *4* the black PVC top plate. **b** The Phenovator system in action: *5* the imaging head carrying the camera (the *red* light is the saturating pulse for ΦPSII measurement), *6* the X–Y rails used to position the imaging head

The Phenovator we designed consists of five main parts: a supporting frame, an ebb and flood hydroponic system, an XY camera movement system, a camera and a computer to control camera movement, imaging and data storage (Fig. 2). The supporting frame was constructed from 100 × 100 mm^2^ box-section aluminium beams (www.maytec.org) to support X–Y rails and the basins in which plants were grown. It also provides the rigid, stable camera platform necessary for imaging. To be able to image quickly requires a platform that is sufficiently stiff to eliminate vibration after the camera movement is complete. The camera movement system (www.elmekanic.nl) is capable of speeds of 6 m s^−1^ (though for safety reasons this is currently limited to only 1 m s^−1^) and allows high reproducibility of camera positioning. We use a so-called “ebb and flood” hydroponic irrigation system to water and feed the plants growing in a rockwool (www.grodan.com) substrate (Fig. 2a). Rockwool is a synthetic, relatively inert, fibrous substrate which allows manipulation of plant nutrition regimes [18].

The growth system (Fig. 2) is comprised of two irrigation basins, each with their own irrigation tank and pump, which allows for two different irrigation and nutrient regimes to be applied in the same experiment. Each basin has space for 720 rockwool blocks 40 × 40 × 40 mm in size giving a total capacity of 1440 rockwool blocks. The rockwool blocks are held 20 mm apart by a PVC grid that is attached to a rigid stainless steel grid upon which the blocks rest. The PVC grid prevents any sideways movement of the rockwool blocks which could cause the plants to shear, and is held 15 mm above the stainless steel base grid by spacers. The stainless steel grid provides structural integrity to the rockwool support system and is supported 5 mm above the bottom of the irrigation basin. The perforations in the grid allow for free circulation of nutrient solution, ensuring that all blocks receive irrigation for approximately the same amount of time. A spacing of <5 mm between the stainless steel base and the irrigation basin was found to sometimes cause problems of root death, possibly due to trapping of nutrient solution and anaerobiosis. On top of the rockwool blocks there is a black plastic non-reflective sheet of foamed PVC, 3 mm thick (Figs. 2a, 4). In this sheet, 3-mm countersunk holes were drilled at distances of 60 mm and positioned above the centre of each rockwool block. All three layers are held in place using threaded stainless steel pins which were welded to the stainless steel grid. Four support studs fit into sockets drilled into the irrigation basin to hold this grid in a fixed position. All materials were tested for phytotoxicity and corrosion resistance, and were washed thoroughly before use. The black plastic cover ensures that there is no algal growth, restricts soil dwelling organisms such as the larvae of fungus gnats (*Bradysia* spp.) and minimises background noise in the images, making automated image processing much easier.

Images are recorded using a monochrome camera (Pike; www.alliedvisiontec.com) mounted on the X–Y movement system. An eight-position filter wheel is mounted between the lens and the ccd chip of the camera to capture images in different wavelength bands. We measure reflectance at 480, 532, 550, 570, 660, 700, 750 and 790 nm with each filter having a full width at half maximum (FWHM) of 10 nm; these narrow spectral wavelength measurements allow for estimation of a range of plant pigments. The reflection bands at 480, 570 and 660 nm are used to construct red, green and blue (RGB) colour images. Chlorophyll content (Chl) is estimated from reflectance (R) at 700 and 790 nm after AA Gitelson et al. [20] Chl = (R700^−1^−R790^−1^) × R790.

Projected leaf area (PLA) provides a good estimate of above ground biomass [29] and is estimated from near infrared (NIR) reflection at 790 nm; this wavelength was chosen so the plants could be measured both day and night without disturbing the day-night cycle. Four NIR light emitting diodes (LED) with a FWHM of 40 nm and a maximum radiant power of 1 W per LED provide the 790 nm radiation. NIR measurements are taken every 3 h resulting in eight images per day.

We use chlorophyll fluorescence imaging to measure Φ_PSII_ (the light-use efficiency of PSII electron transport, also known as Fq′/Fm′, or ΔF/Fm) [3, 16, 41] using a variation of the method of Genty and Meyer [17]. This method has the advantage of a good signal to noise ratio and has proved very suitable for our imaging conditions in which the unfiltered background irradiance is low owing to the shadowing effect of the imaging system. Measurements are made by illuminating the plants at the growth chamber actinic light level (200 or 550 µmol m^−2^ s^−1^) with a centre wavelength of 630 nm and a FWHM of 20 nm for 10 s followed by a 2 s saturating pulse of 5000 µmol m^−2^ s^−1^ using LEDs attached to the Phenovator camera head. At the end of the 10 s of actinic light and prior to the saturating light, 24 images are taken and averaged to generate the Fs image. During the saturating light pulse six images are taken of which that with the highest signal is used for the Fm′ image. The LEDs are turned off after the saturating pulse and an additional 24 images are taken and averaged in order to generate a dark image to account for any background light from the fluorescent lamps in the growth chamber. A fluorescent target, applied as a rubber compound [purchased from Thorlabs (www.thorlabs.de), but since withdrawn from the market] that shows fluorescence over a wide range of wave lengths, is imaged at the beginning of each measurement sequence in order to provide a factor to correct the Fs and Fm′ measurements for the difference in light intensity used to produce the images [17]. The camera measurement scheme was programmed so that immediately neighbouring positions were skipped and returned to later, thus allowing time for any disturbance of adjacent plants by either an increase or decrease of their irradiance to dissipate. Thus the Phenovator comprises only four moving parts, the X movement system, the Y movement system (these both comprise of motors, drive belts and bearings), the filter wheel and the camera focus. This simplicity is a strong advantage when long term experiments are undertaken.

### Data processing

The growth platform containing the 1440 plants is divided up into 120 imaging positions (Additional file 1: Figure S1) each of which contains 12 plants (3 × 4) thus each measurement cycle results in 120 images each containing 12 plants. Different measurement tasks (imaging Φ_PSII_, NIR reflectance or spectral imaging) can be programmed in a daily schedule, which is used over the entire experiment. Analysis software has been developed to convert raw images from the imaging system to images of physiological parameters (e.g. Φ_PSII_) or biochemical composition (e.g. chlorophyll content). Each image is matched to a table position, and the genotype planted at each position is provided via a comma separated (csv) file, thus enabling the image processing software to group images by genotype. Based on images containing 12 plants the analysis software (available upon request) calculates per replicate the parameters for each genotype. Each measurement protocol (e.g. measurement of Φ_PSII_) produces its own parameters, which are calculated from a selected area within the image using a mask derived from the desired plant. A grid of vertical and horizontal reference lines (shown in Additional file 1: Figure S1) is set by the user and provide the coordinates around which a box is drawn to select individual plants. A greyscale threshold (or mask), set by the user, is used to distinguish the plant from the background within this box. Twelve areas are defined and used to obtain a specific plant from the image. Only the pixels within the mask are used to estimate the phenotypic parameters.

All images (raw data and derived data) are stored, and the values of each phenotype are calculated per pixel. Both the pixel values and the averages over images are available to output in csv format. The spatial distribution of pixel data within any stored image can be shown (Fig. 1; Additional file 1: Figure S1). Since our plants were grown for only 4 weeks under non-stressful conditions there was no spatial variation in any parameter so we will not discuss this further.

## Results

### System uniformity

The (spatial) uniformity and (temporal) reproducibility of the system were assessed by estimating the magnitude of several design factors using a mixed model (see “Methods” section; Additional file 2: Appendix S1 for an overview of the experiments), which included random effects for genotype, experiment, basin, and table position (Additional file 3: Appendix S2; Additional file 4: Data S1, Additional file 5: Data S2). Using this model, genotypic means were calculated as the best linear unbiased estimators (BLUEs) for genotype. Spatial variability was modelled by row (*x*) and column (*y*) effects, as well as within image rows *x*_*within*_ and columns *y*_*within*_. While *x* and *y* modelled the coordinates across the whole platform, *x*_*within*_ and *y*_*within*_ modelled the spatial effects within images of 12 plants (3 × 4). In addition to the main effects, second and third order interactions between design factors were included. A more detailed description of all design factors is given in Additional file 3: Appendix S2.

For all traits and time points there is considerable genetic variation: the variance component for genotype is of a similar order of magnitude as the residual error variance, which is consistent with the heritability estimates found below. Although the main effect of experiment was substantial, the genotype by experiment interaction was negligible for almost all traits. Only for the spectral measurements at 700 and 750 nm the genotype by experiment interaction was larger, but still small compared to the main genotypic variance (Additional file 4: Data S1, Additional file 5: Data S2). The phenotypic ranking of the genotypes can therefore be expected to be consistent across experiments. For Φ_PSII_ and spectral measurements, the position within the image showed a considerable main effect which is likely due to light gradients in the camera head. This effect of position within the image showed no interaction with genotype (Additional file 4: Data S1, Additional file 5: Data S2) and thus could be corrected for. In a few cases, there was some interaction between experiment and within image position, but never with genotype. Table position and the x and y coordinate across the whole platform showed a small main effect for some of the spectral measurements. Nevertheless, the very low variances of the interactions between genotype, experiment and the design-effects indicate that we can combine data from different experiments, allowing phenotyping of potentially thousands of genotypes.

### Phenotypic variation

**Fig. 3:**
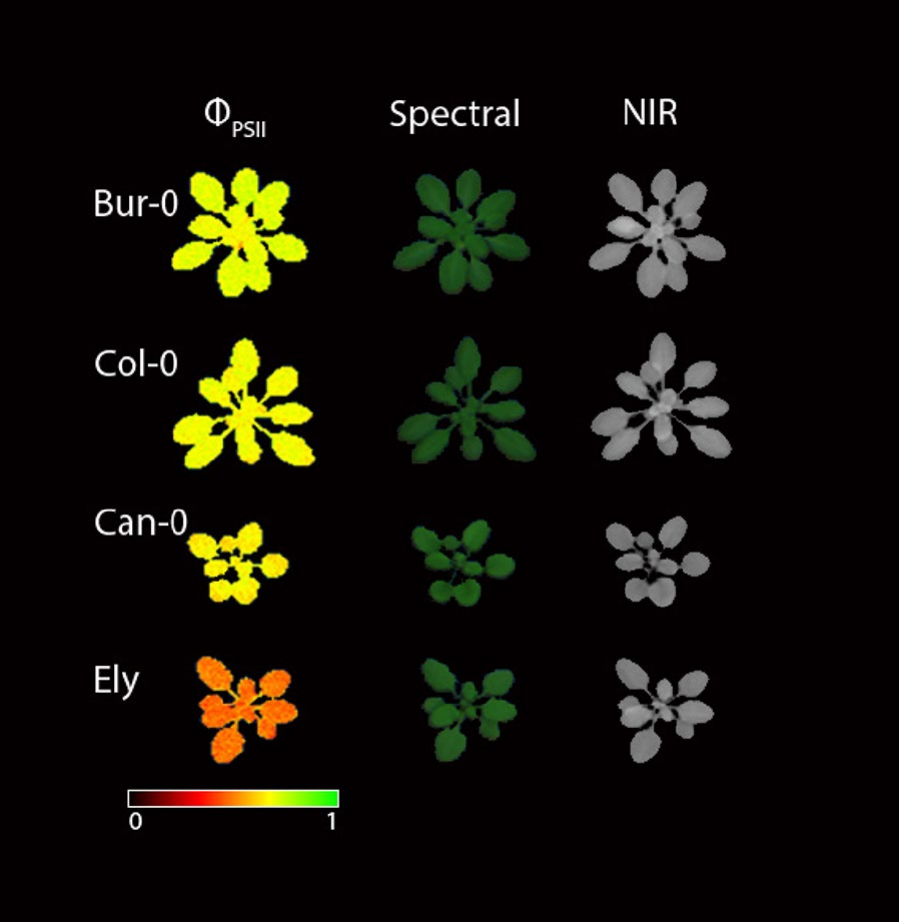
Examples of images generated by the Phenovator. The *first column* shows false colour images of photosystem II efficiency (ΦPSII) running from 0 (*black*) to 1 (*green*). The *second column* shows the red–green–blue (RGB) output of the spectral measurements. The *third column* shows the images generated by near infrared imaging (NIR) at 790 nm. The *rows* correspond to four different genotypes, accessions Bur-0, Col-0, Can-0 and Ely. Ely is atrazine resistant, hence the much lower ΦPSII

The Phenovator has three main imaging protocols in routine use (Fig. 3). The first is used to measure photosynthetic efficiency via chlorophyll fluorescence (Φ_PSII_), the second is used to measure pigment content via spectral imaging and the third measures PLA via NIR imaging.

**Fig. 4:**
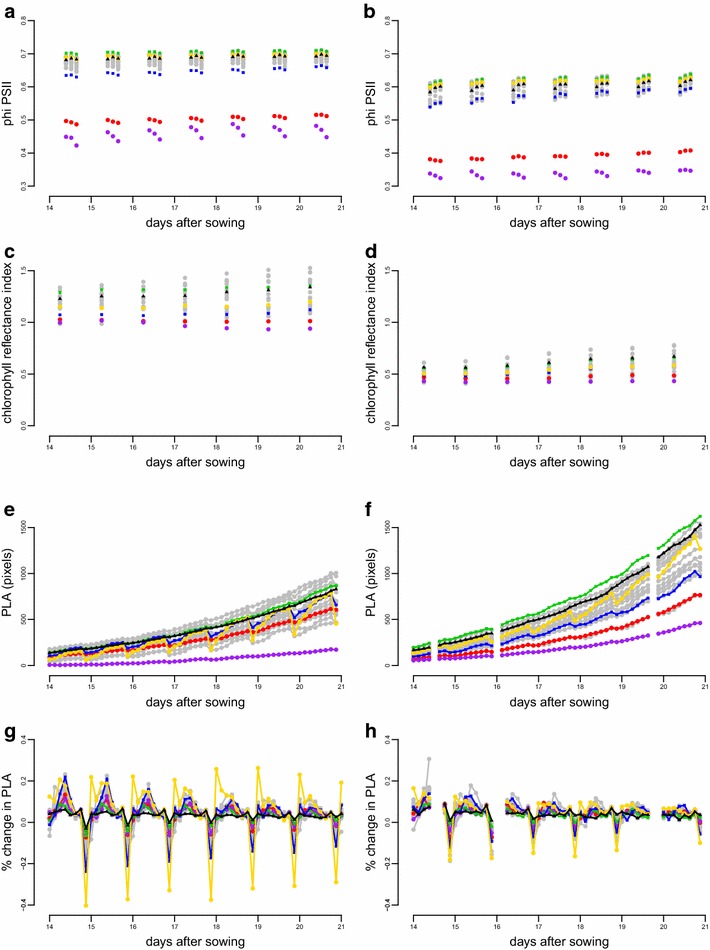
Phenotypic diversity in twenty Arabidopsis genotypes grown at 200 µmol m^−2^ s^−1^ light intensity (**a**, **c**, **e**, **g**), and 550 µmol m^−2^ s^−1^ light intensity (**b**, **d**, **f**, **h**). Graphs (**a**, **b**) shows Φ_PSII_ through time; **c**, **d** shows chlorophyll reflectance index; **e**, **f** shows projected leaf area (PLA). Finally, **g**, **h** shows percentage change in PLA every 3 h. All data points are genotypic means (BLUEs), combining observations on replicates from different experiments into one representative value for each genotype at each time point. Six genotypes, An-2 (*yellow circles*), BC354 (*purple circles*), Bur-0 (*green squares*), Col-0 (*black triangles*), Ely (*red circles*) and Ts-1 (*blue squares*) are indicated in *colour*. *Error bars* have been excluded for clarity, the significance of between genotype differences is apparent from the heritability estimates in Fig. 6

Figure 4a, b shows the total variation for Φ_PSII_ for 20 genotypes grown at 200 and 550 µmol m^−2^ s^−1^ light intensity. The two different light intensities were chosen both to test the flexibility of the system and to assess the response of the genotypes to these different conditions. Φ_PSII_ is influenced by both the light intensity and the genetic background of the plant measured. In addition to these differences, the high resolution measurements allow the observation of both a daily fluctuation in Φ_PSII_ as well as a gradual upward trend through time at the higher light intensity.

The two genotypes with the lowest Φ_PSII_ are Ely, an atrazine resistant accession known to have a low light-use efficiency for PSII electron transport [11], and RIL BC354 from the Bur-0 × Col-0 population [38], which is known to carry a mutant version of the *PDE237* gene affecting photosynthesis [43]. However, even without these unusual genotypes there is substantial variation for Φ_PSII_. The variation from approx. 0.62 to 0.72 at 200 µmol m^−2^ s^−1^ and 0.54 to 0.63 at 550 µmol m^−2^ s^−1^ is about 12 % (assuming an upper limit of 0.8 for Φ_PSII_) for normal natural accessions, extending to almost 40 % when the lines with unusually low light-use efficiency are included.

**Fig. 5:**
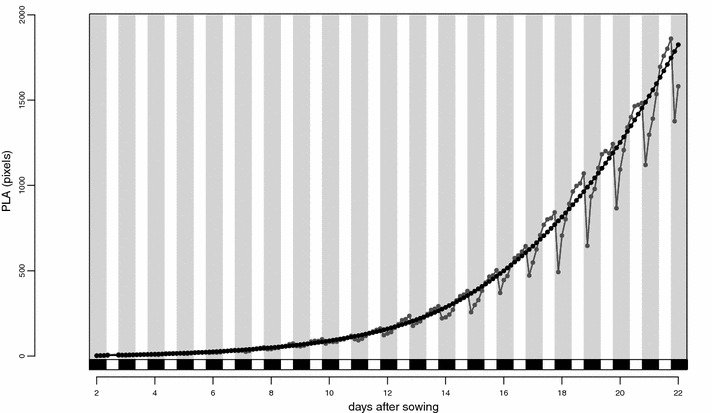
Curve parameterisation of projected leaf area (PLA) for one replicate of genotype An-2. *Grey line* and *filled squares* show raw data, while *black line* and *filled circles* show fitted values; *white* and *grey bars*, projected in *white* and *black* on the X-axis, indicate the day-night cycle

The chlorophyll reflectance index (Fig. 4c, d) is a linear measure of chlorophyll content and, as expected [1], decreases as the irradiance is increased from 200 to 550 µmol m^−2^ s^−1^. The phenotypic variation in spectral reflectance at each wavelength can be observed, with an increase in light intensity having opposite effects on different wavelengths (Additional file 6: Figure S2). PLA can be measured at short intervals, allowing the construction of growth curves (Fig. 4e, f). Both genetic background and light intensity have a large effect on growth rates. Another interesting phenomenon is the undulating nature of the curves due to leaf movement. The percentage difference between images at neighbouring time points shows the movement more clearly (Fig. 4g, h). The plant growth and leaf movement phenotypes are easily revealed and analysed because of the high imaging frequency. The fluctuation in PLA due to leaf movement can result in negative apparent growth rates, so we smoothed the curves before estimating growth rates (Fig. 5).

### Genetic variation

**Fig. 6:**
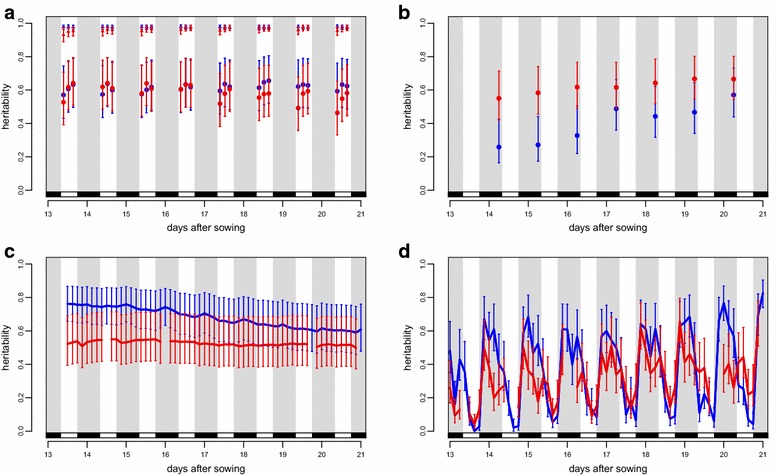
Time course of heritability. **a** Heritabilities for ΦPSII at two light levels with and without extreme genotypes. *Blue circles* show heritabilities of ΦPSII at 200 µmol m^−2^ s^−1^ without considering Ely and BC354. *Blue triangles* show heritability of ΦPSII at 200 µmol m^−2^ s^−1^ including data for Ely and BC354. *Red data point* as for *blue* but where plants were grown at 550 µmol m^−2^ s^−1^. **b** Heritability of chlorophyll reflectance. **c** Heritability of projected leaf area (PLA). **d** Heritability of percentage change in projected leaf area. 200 µmol m^−2^ s^−1^ (*blue*) and 550 µmol m^−2^ s^−1^ (*red*), *white* and *grey bars* indicate the day night cycle. *Error bars* are 95 % confidence intervals

The heritability of a trait is a measure of the proportion of phenotypic variance explained by genetic effects [42]. Figure 6a shows the heritability through time for Φ_PSII_. Addition of the photosynthetic extremes greatly inflates the genotypic variance which results in very high estimates of heritability. The heritability of Φ_PSII_ also shows a slight but recurrent daily rise, but is not affected by the difference in light intensity. The heritability of chlorophyll reflectance index and PLA show more gradual changes through time and are different depending of the light intensity (Fig. 6b, c). Heritability of percentage change in PLA on the other hand is much more dynamic, with values shifting from 0.04 to 0.83 in the course of 6 h (Fig. 6d), emphasizing the importance of frequent measurements. In general the heritability was slightly lower at higher light intensity, probably due to reduced overall leaf movement (Fig. 4g, h). The most pronounced fluctuation is between day and night with heritability being much higher in the night than during the day. For spectral reflectance and growth curve traits the heritability also shows variation through time but in a less dynamic fashion, shifting over the course of several days (Additional file 7: Figure S4; Additional file 8: Figure S5).

## Discussion

### Uniformity and reproducibility

Uniformity and reproducibility of the Phenovator is essential if it is to be of any use. Although some design factors had a considerable main effect on the measurement (see Additional file 4: Data S1, Additional file 5: Data S2), the interaction of these design factors with genotype was very small and can be corrected for. The effect of the position within the image can be attributed to light gradients in the camera head, while the effect of experiment may be due to small accumulated differences which are collectively significant but individually minor [32]. The correction for design factors is achieved through the calculation of BLUEs for each genotype (Fig. 4). The ability to correct design factors greatly increased the signal-to-noise ratio of the Phenovator, with signal being the genotypic effect and noise being unexplained phenotypic variation. This will be important when screening genetic mapping populations, as a larger contribution of the genotypic effect to the signal will increase the heritability of the measured trait. Finally, the estimation of the effect of experiment and its negligible interaction with genotype or other design factors allowed the combination of data from different experiments, greatly increasing the effective capacity of the system and the power of our statistical analysis.

### Phenomic data

The only comparable system measuring photosynthetic and growth parameters is the GROWSCREEN FLUORO [23], which can phenotype up to 60 plants h^−1^ for growth and dark-adapted (maximum) PSII efficiency (Fv/Fm). Our system can measure the PLA of 1440 plants in 20 min, and their light-adapted PSII efficiency, or operating efficiency (Φ_PSII_, Fq′/Fm′) [3], in less than an hour. The operating efficiency of Φ_PSII_ directly relates to the rate of carbon fixation and ultimately growth and thus is physiologically more relevant than Fv/Fm when assessing genotype performance in a range of conditions [16]. Using measurements of Φ_PSII_ we were able to determine differences in the rate of photosynthesis and estimate the genetic contribution to these differences for 20 genotypes of Arabidopsis grown at two light levels (Figs. 4a, b, 6). Of interest is the daily rise in Φ_PSII_ for all genotypes at 550 µmol m^−2^ s^−1^ with the exception of RIL BC354, which shows a daily decline in Φ_PSII_. The mutant allele of *PDE237* (At4g30720), normally encoding an oxidoreductase/electron carrier residing in the chloroplast stroma [43], probably affects Φ_PSII_ due to accumulated PSII damage during the day.

Using NIR light allowed us to measure PLA throughout the day and night without disturbing the photoperiod. Since one NIR measurement of all 1440 plants takes only 20 min we could measure all plants 72 times per day. This frequency exceeds that required to capture growth or leaf movement in most cases, but it could be valuable to capture rapid responses such as those induced upon water stress or disease infection. For measurements of growth or leaf movement under non-stressed conditions, imaging once every 3 h has proven to be sufficient (Figs. 4g, h, 5).

In addition to our priorities of measuring plant growth and photosynthesis we also measured the reflectance of individual plants at eight wavelengths of light. This made spectral imaging and estimating pigment content possible, which was also highly reproducible across experiments and genotypes. We were able to show a decline in chlorophyll reflectance when the plants were grown under high light conditions (Fig. 4c, d) which is expected according to literature [1].

### Heritability through time

Using the phenotypic values for all traits across all genotypes we calculated the broad sense heritability of the different traits. As expected, heritability was trait specific, reflecting the genetic variation present for the trait. An unexpected finding was the amount this could vary through time. Daily fluctuations in heritability for some traits ranged from 0.04 to 0.83 (Fig. 6). As far as we know it is the first time this has been described in such detail, which is the consequence of imaging at such high frequency. We would never have detected this if images were taken at single or irregular time points per day. The magnitude and frequency of this variation in heritability was much greater than expected and strongly argues for high-frequency measurements. In the case of percentage change in PLA every 3 h, reflecting leaf angle at different time points (Fig. 6b), the fluctuations in heritability show a diurnal pattern with a recurrent decline during the day under both light intensities. This may indicate the higher selection intensities present for leaf angle in light than in the dark, or alternatively a wider range of optima for leaf angle in the dark. In this case the measurement frequency was sufficient to capture changes in leaf movement using a simple difference method (the step change in PLA). This is apparent as an episodic, daily event. Note that this simple difference method captures the change in PLA due to leaf movement, but not that due to growth. When the measurements are viewed collectively, growth is nonetheless apparent (Fig. 5).

Two recent studies in Arabidopsis used high-throughput phenotyping to describe changing heritability through time. The first showed changes in the heritability for rosette compactness, which appears to increase linearly until the rosette has fully formed [46]. Two other traits, rosette area and circular area, showed fewer changes. The second study focused on root gravitropism [33]; after being reoriented by 90°, roots of seedlings were imaged every 2 min for 8 h. The gravitropic response also showed a change in heritability through time. Interestingly some of the QTL underlying this changing heritability were time specific and only detectable for short periods.

Our results show both large and dynamic fluctuations in heritability due to changes in the relative contribution of genetic diversity to the traits at different time points (Fig. 6; Additional file 7: Figure S4; Additional file 8: Figure S5). The implications of this dynamic variation in heritability are wide ranging. For traits with such strong fluctuations in heritability, the time they are measured at will have a considerable impact on the extent of variation found. For crop breeding programs this could result in the fixation of alleles which may not be optimal for trait improvement. Screening when heritability is low will reduce the ability to detect genetic variation and the response of the germplasm to selection is likely to be curtailed [42]. This can lead to a waste of resources in large-scale breeding experiments. Awareness of the fluctuations in heritability can also be used to inform the breeder when the variation in phenotypes is most relevant. As shown by Moore et al. [33] the genetic loci responsible for the changing heritability can change through time. If fixation of a specific locus or set of loci is required, then identification of the time when they contribute most to phenotypic variance will result in more targeted breeding, and again, greater efficiency. Awareness of the extent and time dependency of variation in heritability will thus maximise the return on investment in trait selection [4].

From an ecological and evolutionary perspective, stronger selection often results in reduced heritability [34], thus if the intensity of selection varies with developmental time, traits which contribute to fitness when selection is greatest are likely to show a reduction in heritability. While this will require further validation it illustrates the value of high-throughput phenotyping for generating insights into the genetic architecture of traits and the uses of such insights in the fields of breeding and evolutionary ecology.

## Conclusion: where next?

The objective of our work has been to develop a high throughput phenotyping platform for photosynthesis (Φ_PSII_) and growth. The rationale behind this is that phenotyping advances are essential for further rapid progress in plant genetics and breeding [15, 22, 37]. The choice of photosynthesis and growth was key, as they are both important traits with a complex polygenic architecture, and reliable high throughput phenotyping methods are needed if we are to mine natural variation or induced mutant libraries for these traits. Photosynthesis is of particular importance as it is the only major physiological trait not to have been directly bred for, and thus represents uncharted territory within which there may be considerable scope for crop improvement [14, 30]. In nature photosynthesis has been shaped by selection in environments where many resources are limited but the supply of fixed carbon is not usually a limiting factor for growth [26], while in agriculture, resources are more abundant and the supply of fixed carbon is often limiting [35]. Adaptations which evolved to increase survival in the wild, but reduce yield in an agricultural context, may be selectively removed [8]. For any such breeding program to be a success, there needs to be appropriate phenotyping [6]. We have proved this is possible for Arabidopsis, though the system we describe would be suitable for any species which forms a flat rosette and for seedlings of most other species. Besides their importance for crop improvement, high throughput phenotypers are essential for quantitative genetic studies such as QTL or GWA mapping. High throughput screening will aid forward genetics approaches for the identification of QTL and the genes responsible for the phenotypic differences in a population [37, 40]. This is especially relevant when looking at natural accessions as such differences may represent adaptive alleles increasing fitness under specific environmental conditions [40]. Identification of such alleles is of interest for evolutionary biology and ecology, and to plant breeding as a source of genetic adaptations, which can be used to tailor crop varieties to specific conditions.

The stability and design of the system allowed the combination of data from multiple experiments, increasing the effective capacity beyond the 1440 plants which can fit in a single screen. The design is such that a range of environmental variables, such as temperature, humidity and nutrient availability, can be controlled both across and during experiments. To illustrate this flexibility we conducted one experiment at a higher light intensity. Such variation in the growth environment can be used to uncover hidden genetic variation not expressed under control conditions and identify genes important for adaptation to environmental fluctuations [19].

Phenomic data is also essential for the advance of the omics revolution. To put all the current omics technologies into context, whole plant phenotyping of morphological and physiological traits is necessary. Without such phenomic data the relevance of variation in gene expression, metabolite or protein abundance to plant performance is much more difficult to assess. The integration of all levels of omics data from gene expression to growth rate will allow a systems biology approach to be undertaken which should greatly further our understanding of plant biology [7, 24, 45]. Our data show how informative phenomics data can be, revealing, for example, how a basic genetic parameter such as heritability can vary through time. This insight is a direct result of the expanded throughput, and particularly, intensity of measurements. The level of accuracy and throughput of our system shows it to be ideally suited for screening large populations of plants thus allowing future quantitative genetic studies of photosynthesis, growth, and the response of these traits to a range of environmental perturbations in Arabidopsis or any rosette species, and thus explore a wide range of dynamic responses of plants, in detail, over time.

## Methods

### Plant material and cultivation

Unless otherwise stated all plants were grown as follows: seed was sown on wet filter paper and stratified for 6 days at 4 °C. After stratification seed was sown directly on wet rockwool (www.grodan.com) which had been pre-soaked in a nutrient solution designed for Arabidopsis (see Additional file 9: Table S1 for composition). One seed was sown per rockwool block (system described in “System design” section). The growth conditions were as follows, 10/14 h day/night, irradiance normally 200 µmol m^−2^ s^−1^, and 550 µmol m^−2^ s^−1^ in the high light experiment, 20/18 °C day/night temperature, 70 % relative humidity, and ambient CO_2_. Plants were irrigated daily with nutrient solution for 5 min. In total 57 genotypes were screened across four experiments, see Additional file 2: Appendix S1 for details of genotype identity and number of replicates. The Φ_PSII_ estimates were compared with those of a MINI-PAM fluorometer (www.walz.com) to validate the measurements and no significant differences were found.

### Measurement protocols

Φ_PSII_ was measured daily, 1, 4 and 7 h into the photoperiod. This was considered sufficient to document any variations in the phenotype and allowed time for other measurements such as NIR, which was measured every 3 h.

### Statistical analysis

#### Variance components

The importance of several design factors was assessed by fitting the following mixed model for each trait and time-point using asreml-R [5]:

1 $$\begin{aligned} Y & = \mu + C + G + Exp + Basin + x + y + TablePosition \\ & \quad + x_{within} + y_{within} + G \times Exp + Exp \\ & \quad \times \left( {Basin + x + y + TablePosition + x_{within} + y_{within} } \right) \\ & \quad + Exp \times Basin \times G + Exp \times G \times \left( {x_{within} + y_{within} } \right) + R(Error) \\ \end{aligned}$$

where µ is the overall mean, and G, Exp and Basin are the factors for respectively genotype, experiment and basin. The factor C represents check-genotypes that were not included in subsequent analyses, but included in the mixed model in order to better estimate the variance components; it has one level for each check-genotype and one additional level representing all other genotypes. All terms except µ and C are defined as random effects. For traits and time-points that were only present in a single experiment, all terms involving Exp were dropped from the model. Spatial variability was modelled by the factors *x*, *y*, TablePosition, *x*_*within*_ and *y*_*within*_ which represent respectively rows, columns, table (camera/image) position and within image rows and columns. While x and y model the coordinates across the whole platform, *x*_*within*_ and *y*_*within*_ model the spatial effects within images of 12 plants (3 × 4). A more detailed description of all design factors is given in Additional file 3: Appendix S2.

#### Genotypic means

Genotypic means used in Fig. 4 were calculated as the best linear unbiased estimators (BLUEs) for genotype, using a mixed model identical to Eq. (1) but with genotype as fixed effect.

#### Heritability estimates

Defining and estimating heritability in the context of a mixed model as defined by Eq. (1) is known to be difficult, since not only the residual error contributes to the environmental variance (the generalized heritability proposed in Oakey et al. [36] concerns line heritability and not the (plot level) heritability $\sigma_{G}^{2} /(\sigma_{G}^{2} + \sigma_{E}^{2} ),$ which is of interest here). To obtain more interpretable and commonly used heritability estimates we therefore performed classical analysis of variance (ANOVA) for the linear model with (fixed) effects for genotype, basin nested within experiment, and within image *x*_*within*_ and *y*_*within*_ coordinates. This included the most important main effects identified by the mixed model analysis described above; the fact that the interactions of design factors with genotype were small, justifies the effects being fixed here. The genetic and environmental variance were estimated by respectively $(MS(G) - MS(E))/\bar{r}$ and MS(E), where MS(G) and MS(E) are the mean sums of squares for genotype and residual error [28, 31]. Broad sense heritability was then estimated by the ratio of estimated genetic variance over the sum of estimated genetic and environmental variance. To facilitate direct comparison, heritability was estimated using 20 genotypes which were screened under both light conditions (see Additional file 2: Appendix S1 for details on the genotypes used).

### Growth curve characterisation

PLA was measured throughout each experiment from NIR images and the masks generated from the Φ_PSII_ images, a total of 11 images per day. In order to summarize these data and estimate growth rates from repeated plant-size measurements, a flexible curve was fitted to the data for each plant. We used P-splines as a flexible semiparametric description of the curves [10]. P-splines are penalized B-splines resulting in smooth piecewise polynomial curves. For the implementation in the context of this paper we used the R package mgcv [44] with the function gam with its option for P-splines. Fitted curves and addition growth parameters are plotted in Fig. 5 and Additional file 10: Figure S3. The (empirical) slope at all time points is calculated directly from the fitted values of the curve. Relative growth rates can be calculated based on the raw data series. However, for fluctuating time series growth rates are more reliable when a smooth curve is base of their calculation (Additional file 10: Figure S3).

## Additional files

10.1186/s13007-016-0113-2 Print screen showing analysis software. The top left panel shows all 120 imaging positions in green with the positions of the four replicates highlighted in yellow. These images of 12 plants are shown in the top row of pictures headed Rep A to Rep D. The plant which corresponds to the genotype being analysed is surrounded by a red box in each image. This plant is cut from the image using a mask level set in the control panel which is shown at the bottom of the image. The resulting image is thing shown by the middle row of pictures. Note this shows a pixel map of ΦPSII distribution. This pixel map is then plotted as a histogram for each image in the last row of pictures.
10.1186/s13007-016-0113-2 Overview of experiments conducted and genotypes used. A list of aliases and geographical origins is also included as well as the number of replicates sown and the experiments each genotype was included in.
10.1186/s13007-016-0113-2 Description of design factors described in Data S1 and Data S2, calculated across three experiments at 200 µmol m^-2^ s^-1^ and across a single experiment at 550 µmol m^-2^ s^-1^. See Appendix S1 for further details on experiments and number of replicates used.
10.1186/s13007-016-0113-2 Variances of all design factors and interactions for each trait at 200 µmol m^-2^ s^-1^. Separate excel sheets correspond to different traits measured. Raw data and graphs of variance components through time are shown on each excel sheet.
10.1186/s13007-016-0113-2 Variances of all design factors and interactions for each trait at 550 µmol m^-2^ s^-1^. Separate excel sheets correspond to different traits measured. Raw data and graphs of variance components through time are shown on each excel sheet.
10.1186/s13007-016-0113-2 Phenotypic variation in spectral reflectance at eight wavelengths. Phenotypic diversity in twenty Arabidopsis genotypes grown at 200 μmol m^-2^ s^-1^ light intensity (a, c, e, g, i, k, m, o), and 550 μmol m^-2^ s^-1^ light intensity (b, d, f, h, j, l, n, p). Wavelength assessed is indicated on the y axis. All data points are genotypic means (BLUEs), combining observations on replicates from different experiments into one representative value for each genotype at each time point. Six genotypes, An-2 (yellow circles), BC354 (purple circles), Bur-0 (green squares), Col-0 (black triangles), Ely (red circles) and Ts-1 (blue squares) are indicated in colour. Error bars have been excluded for clarity, the significance of between genotype differences is apparent from the heritability estimates in Figure S4.
10.1186/s13007-016-0113-2 Time course of Heritability for spectral reflectance at eight wavelengths. (a) 480 nm, (b) 532 nm, (c) 550 nm, (d) 570 nm, (e) 660 nm, (f)700 nm, (g) 750 nm, and (h) 790 nm. 200 μmol m^-2^ s^-1^ (blue) and 550 μmol m^-2^ s^-1^ (red), white and grey bars indicate the day night cycle. Error bars are 95% confidence intervals.
10.1186/s13007-016-0113-2 Time course of heritability of growth curve parameters for plants grown at 200 µmol m^-2^ s^-1^ light intensity. (a) Projected leaf area (PLA) from near infrared (NIR) measurements. (b) Data from (a) fitted to a curve. (c) The empirical slope of the growth curve and (d) the relative growth rate. White and grey bars indicate the day/night cycle. Error bars are 95% confidence intervals.
10.1186/s13007-016-0113-2 Nutrient solution composition.
10.1186/s13007-016-0113-2 Phenotypic variation in growth curve parameters. Phenotypic diversity in twenty Arabidopsis genotypes grown at 200 μmol m^-2^ s^-1^ light intensity. (a) Projected leaf area (PLA) from near infrared (NIR) measurements. (b) Data from (a) fitted to a curve. (c) The empirical slope of the growth curve and (d) the relative growth rate. All data points are genotypic means (BLUEs), combining observations on replicates from different experiments into one representative value for each genotype at each time point. Six genotypes, An-2 (yellow circles), BC354 (purple circles), Bur-0 (green squares), Col-0 (black triangles), Ely (red circles) and Ts-1 (blue squares) are indicated in colour. Error bars have been excluded for clarity, the significance of between genotype differences is apparent from the heritability estimates in Figure S5.

## Abbreviations

ANOVA: analysis of variance
BLUEs: best linear unbiased estimators
Chl: chlorophyll reflectance
Fv/Fm: dark-adapted (maximum) PSII efficiency
FWHM: full width at half maximum
GWA: genome wide association
LED: light emitting diodes
NIR: near infrared
PLA: projected leaf area
PSII: photosystem II
QTL: quantitative trait loci
RIL: recombinant inbred line
Φ_PSII_: light-adapted PSII efficiency, or PSII operating efficiency
