# Supplementary material for: Phenomics for photosynthesis, growth and reflectance in Arabidopsis thaliana reveals circadian and long-term fluctuations in heritability
Source: Plant Methods. 2016 Feb 15;12:14. doi: 10.1186/s13007-016-0113-y (PMC4754911; doi:10.1186/s13007-016-0113-y)
Supplement: Supplementary file 1 — 10.1186/s13007-016-0113-2 Print screen showing analysis software. The top left panel shows all 120 imaging positions in green with the positions of the four replicates highlighted in yellow. These images of 12 plants are shown in the top row of pictures headed Rep A to Rep D. The plant which corresponds to the genotype being analysed is surrounded by a red box in each image. This plant is cut from the image using a mask level set in the control panel which is shown at the bottom of the image. The resulting image is thing shown by the middle row of pictures. Note this shows a pixel map of ΦPSII distribution. This pixel map is then plotted as a histogram for each image in the last row of pictures. [file 13007_2016_113_MOESM1_ESM.pptx]

## Slide 1
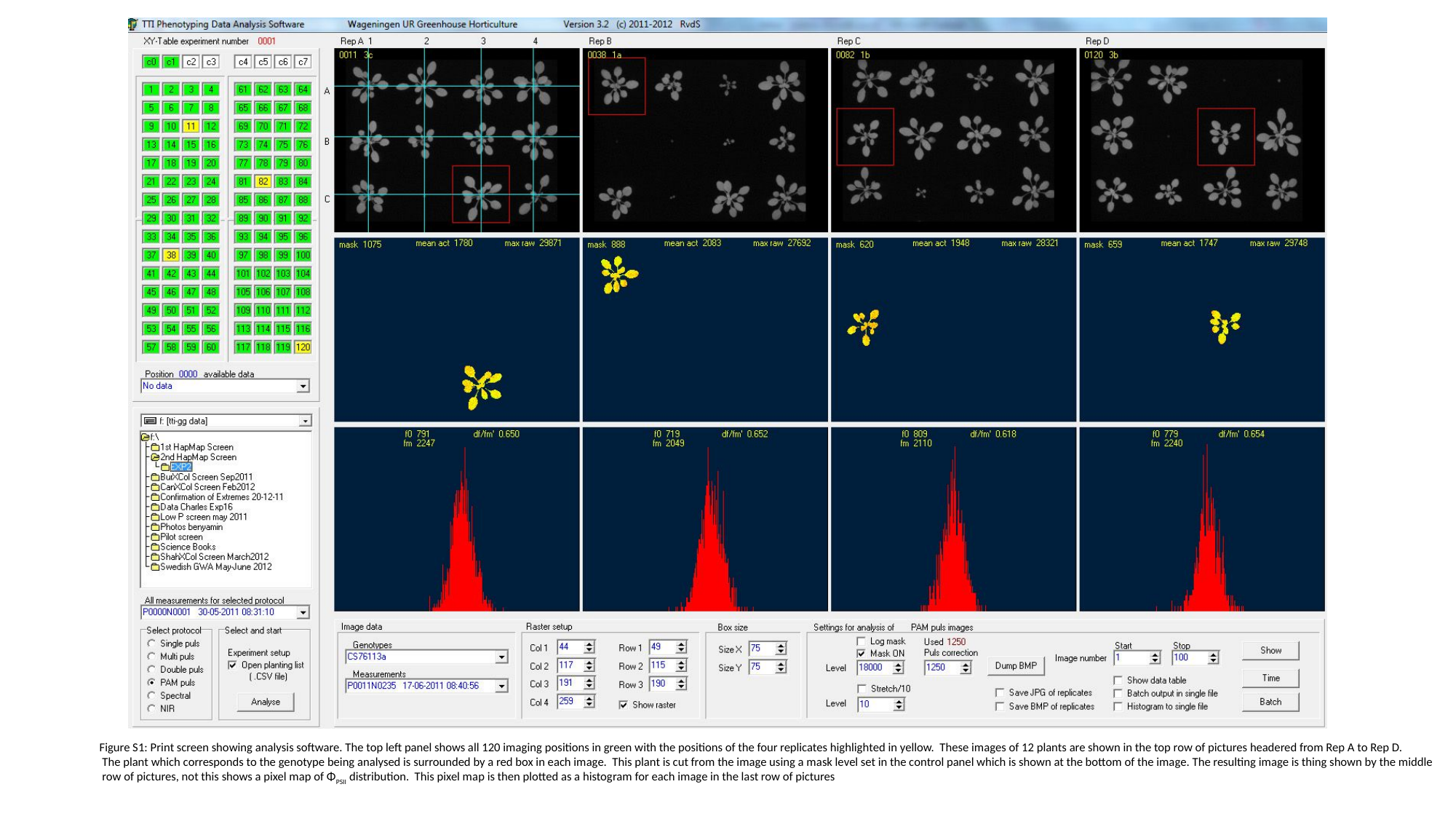

Figure S1: Print screen showing analysis software. The top left panel shows all 120 imaging positions in green with the positions of the four replicates highlighted in yellow. These images of 12 plants are shown in the top row of pictures headered from Rep A to Rep D.
 The plant which corresponds to the genotype being analysed is surrounded by a red box in each image. This plant is cut from the image using a mask level set in the control panel which is shown at the bottom of the image. The resulting image is thing shown by the middle
 row of pictures, not this shows a pixel map of ΦPSII distribution. This pixel map is then plotted as a histogram for each image in the last row of pictures
