# Supplementary material for: Phenomics for photosynthesis, growth and reflectance in Arabidopsis thaliana reveals circadian and long-term fluctuations in heritability
Source: Plant Methods. 2016 Feb 15;12:14. doi: 10.1186/s13007-016-0113-y (PMC4754911; doi:10.1186/s13007-016-0113-y)
Supplement: Supplementary file 3 — 10.1186/s13007-016-0113-2 Description of design factors described in Data S1 and Data S2, calculated across three experiments at 200 µmol m-2 s-1 and across a single experiment at 550 µmol m-2 s-1. See Appendix S1 for further details on experiments and number of replicates used. [file 13007_2016_113_MOESM3_ESM.docx]

**Appendix s2 description of design factors described in Data S1 and Data S2**

Calculated across three experiments at 200µmolm^-2^s^-1^ and across a single experiment at 550µmolm^-2^s^-1^, see Appendix S1 for further details on experiments and number of replicates used.

**Genotype** Main effect of the genetic background on the trait

**Exp** Main effect of experiment on the trait.

**genotype:Exp** Interaction term describing the interaction, if any, between genotype and experiment

**x_within_image** Main effect of the x coordinate within the image of 12 plants taken by the camera

**y_within_image** Main effect of the y coordinate within the image of 12 plants taken by the camera

**Table.pos** Main effect of the imaging position the image of 12 plants was taken at in the table, there were 120 imaging positions.

**X** This is the x coordinate across the whole imaging platform

**Y** This is the y coordinate across the whole imaging platform

**Basin** Main effect indicating which of the two irrigation basin the plant was growing in.

**Exp:Basin** Interaction between basin and experiment

**Exp:Table.pos** Interaction between experiment and imaging position within table

**Exp:x_within_image** Interaction between experiment and the x coordinate within the image of 12 plants taken by the camera

**Exp:y_within_image** Interaction between experiment and the y coordinate within the image of 12 plants taken by the camera

**Exp:x** Interaction between experiment and the x coordinate across the whole imaging platform

**Exp:y** Interaction between experiment and the x coordinate across the whole imaging platform

**Exp:Basin:genotype** Interaction between genotype, experiment, and basin.

**genotype:Exp:x_within_image** Interaction between genotype, experiment, and the x coordinate within the image of 12 plants taken by the camera

**genotype:Exp:y_within_image** Interaction between genotype, experiment, and the y coordinate within the image of 12 plants taken by the camera

**R** residual error
