# Supplementary figures and images for: Phenomics for photosynthesis, growth and reflectance in Arabidopsis thaliana reveals circadian and long-term fluctuations in heritability
Source: Plant Methods. 2016 Feb 15;12:14. doi: 10.1186/s13007-016-0113-y (PMC4754911; doi:10.1186/s13007-016-0113-y)

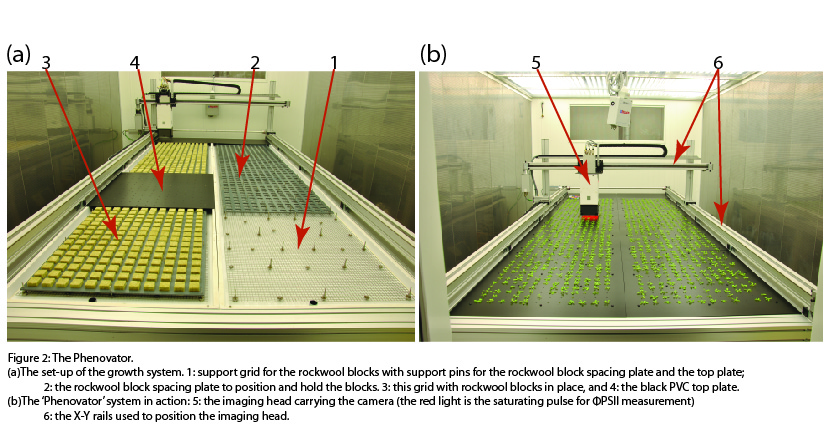

Supplement: Supplementary file 6 — 10.1186/s13007-016-0113-2 Phenotypic variation in spectral reflectance at eight wavelengths. Phenotypic diversity in twenty Arabidopsis genotypes grown at 200 μmol m-2 s-1 light intensity (a, c, e, g, i, k, m, o), and 550 μmol m-2 s-1 light intensity (b, d, f, h, j, l, n, p). Wavelength assessed is indicated on the y axis. All data points are genotypic means (BLUEs), combining observations on replicates from different experiments into one representative value for each genotype at each time point. Six genotypes, An-2 (yellow circles), BC354 (purple circles), Bur-0 (green squares), Col-0 (black triangles), Ely (red circles) and Ts-1 (blue squares) are indicated in colour. Error bars have been excluded for clarity, the significance of between genotype differences is apparent from the heritability estimates in Figure S4. [file 13007_2016_113_MOESM6_ESM.jpg]
