# Supplementary material for: Phenomics for photosynthesis, growth and reflectance in Arabidopsis thaliana reveals circadian and long-term fluctuations in heritability
Source: Plant Methods. 2016 Feb 15;12:14. doi: 10.1186/s13007-016-0113-y (PMC4754911; doi:10.1186/s13007-016-0113-y)
Supplement: Supplementary file 7 — 10.1186/s13007-016-0113-2 Time course of Heritability for spectral reflectance at eight wavelengths. (a) 480 nm, (b) 532 nm, (c) 550 nm, (d) 570 nm, (e) 660 nm, (f)700 nm, (g) 750 nm, and (h) 790 nm. 200 μmol m-2 s-1 (blue) and 550 μmol m-2 s-1 (red), white and grey bars indicate the day night cycle. Error bars are 95% confidence intervals. [file 13007_2016_113_MOESM7_ESM.pdf]

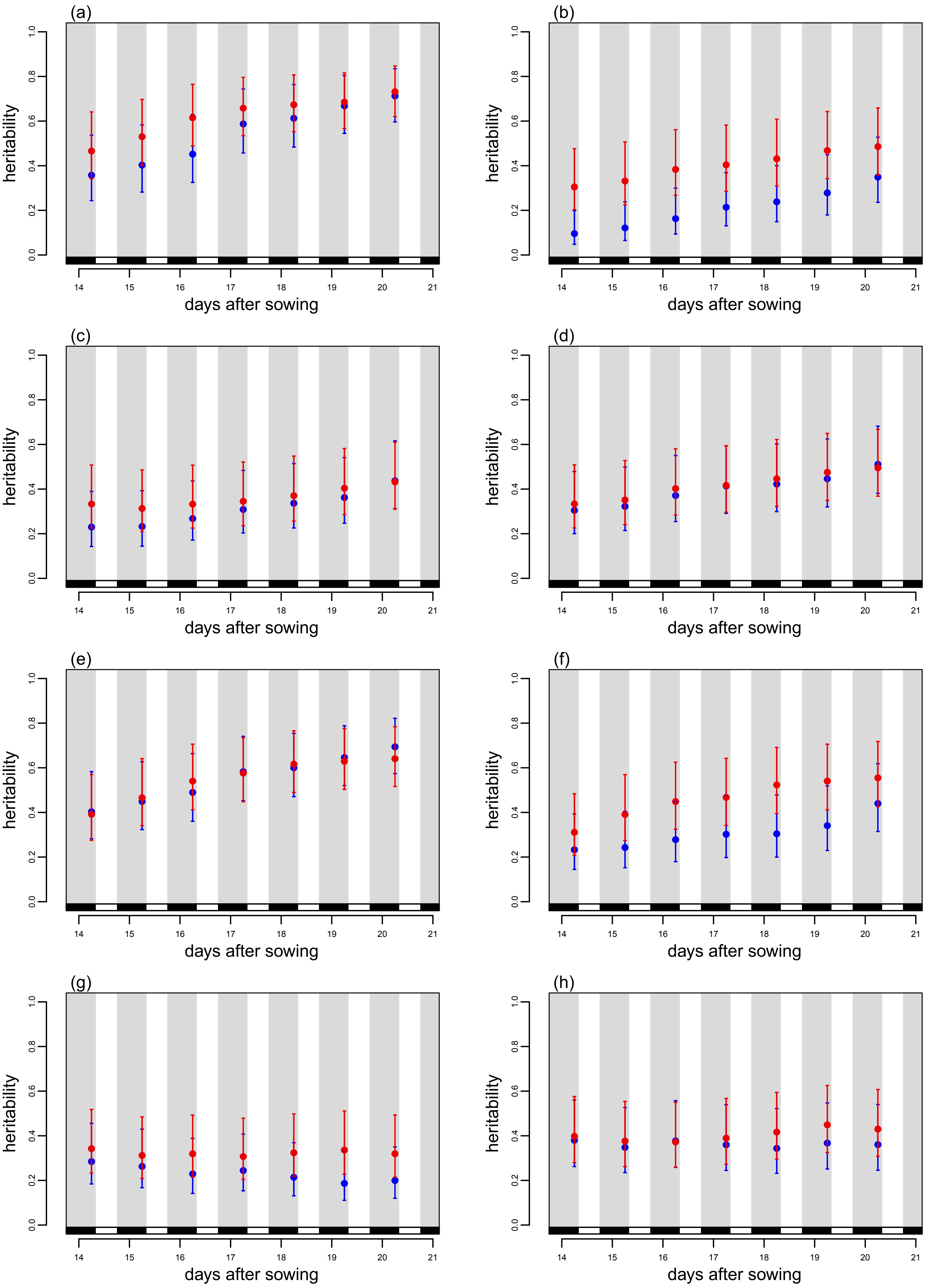

Figure S4. Time course of Heritability for spectral reflectance at eight wavelengths.
(a) 480nm, (b) 532nm, (c) 550nm, (d) 570nm, (e) 660nm, (f)700nm, (g) 750nm, and (h) 790nm.
200µmol m-2 s-1(blue) and 550µmol m-2 s-1(red), white and grey bars indicate the day night cycle.
Error bars are 95% confidence intervals.
