# Supplementary material for: Phenomics for photosynthesis, growth and reflectance in Arabidopsis thaliana reveals circadian and long-term fluctuations in heritability
Source: Plant Methods. 2016 Feb 15;12:14. doi: 10.1186/s13007-016-0113-y (PMC4754911; doi:10.1186/s13007-016-0113-y)
Supplement: Supplementary file 8 — 10.1186/s13007-016-0113-2 Time course of heritability of growth curve parameters for plants grown at 200 µmol m-2 s-1 light intensity. (a) Projected leaf area (PLA) from near infrared (NIR) measurements. (b) Data from (a) fitted to a curve. (c) The empirical slope of the growth curve and (d) the relative growth rate. White and grey bars indicate the day/night cycle. Error bars are 95% confidence intervals. [file 13007_2016_113_MOESM8_ESM.pdf]

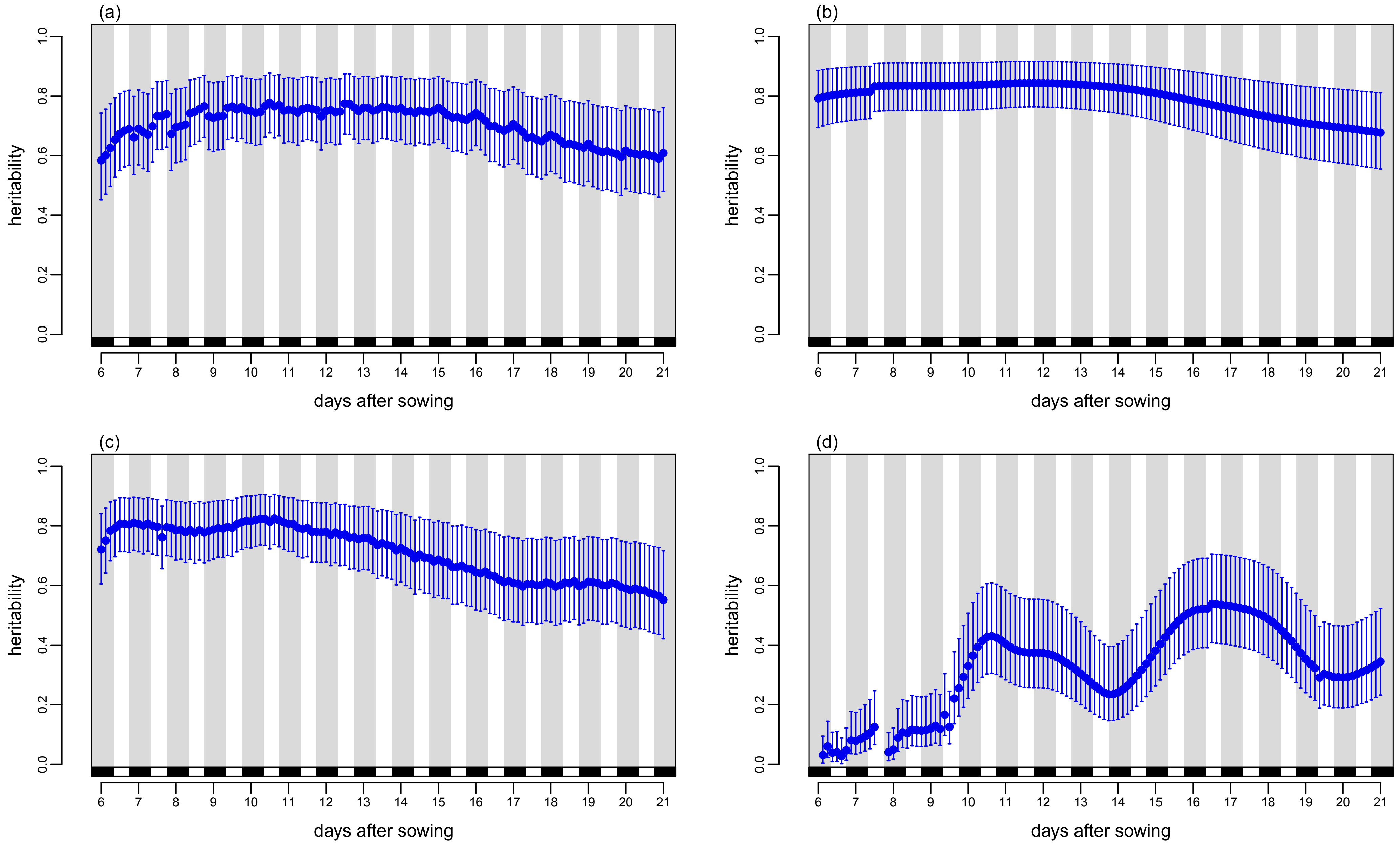

Figure S5. Time course of heritability of growth curve parameters for plants grown at 200 µmol m-2 s-1 light intensity.
(a) Projected leaf area (PLA) from near infra-red (NIR) measurements. (b) Data from (a) fitted to a curve. (c) The empirical slope of the growth curve and (d) the relative growth rate. White and grey bars indicate the day night cycle. Error bars are 95% confidence intervals.
