# Supplementary material for: Phenomics for photosynthesis, growth and reflectance in Arabidopsis thaliana reveals circadian and long-term fluctuations in heritability
Source: Plant Methods. 2016 Feb 15;12:14. doi: 10.1186/s13007-016-0113-y (PMC4754911; doi:10.1186/s13007-016-0113-y)
Supplement: Supplementary file 9 — 10.1186/s13007-016-0113-2 Nutrient solution composition. [file 13007_2016_113_MOESM9_ESM.docx]

**Table S1:** Nutrient solution composition

|  |  | Cations (mmol/l) | | Anions (mmol/l) | | Micronutrients (μmol/l) | | Micronutrients (mmol/l) | |
| --- | --- | --- | --- | --- | --- | --- | --- | --- | --- |
| pH 6.1 | 6.1 | NH_4_ | 1.4 | Cl | 0.2 | Fe | 19 | Si | < 0.01 |
| EC (mS/cm) | 1.5 | K | 5.7 | S | 3 | Mn | 11 |  |  |
|  |  | Na | 0.2 | HCO_3_ | 0.4 | Zn | 6.3 |  |  |
|  |  | Ca | 1.9 | P | 1.24 | B | 22 |  |  |
|  |  | Mg | 1.2 |  |  | Cu | 9.2 |  |  |
|  |  |  |  |  |  | Mo | 0.4 |  |  |
