# Supplementary material for: Phenomics for photosynthesis, growth and reflectance in Arabidopsis thaliana reveals circadian and long-term fluctuations in heritability
Source: Plant Methods. 2016 Feb 15;12:14. doi: 10.1186/s13007-016-0113-y (PMC4754911; doi:10.1186/s13007-016-0113-y)
Supplement: Supplementary file 10 — 10.1186/s13007-016-0113-2 Phenotypic variation in growth curve parameters. Phenotypic diversity in twenty Arabidopsis genotypes grown at 200 μmol m-2 s-1 light intensity. (a) Projected leaf area (PLA) from near infrared (NIR) measurements. (b) Data from (a) fitted to a curve. (c) The empirical slope of the growth curve and (d) the relative growth rate. All data points are genotypic means (BLUEs), combining observations on replicates from different experiments into one representative value for each genotype at each time point. Six genotypes, An-2 (yellow circles), BC354 (purple circles), Bur-0 (green squares), Col-0 (black triangles), Ely (red circles) and Ts-1 (blue squares) are indicated in colour. Error bars have been excluded for clarity, the significance of between genotype differences is apparent from the heritability estimates in Figure S5. [file 13007_2016_113_MOESM10_ESM.pdf]

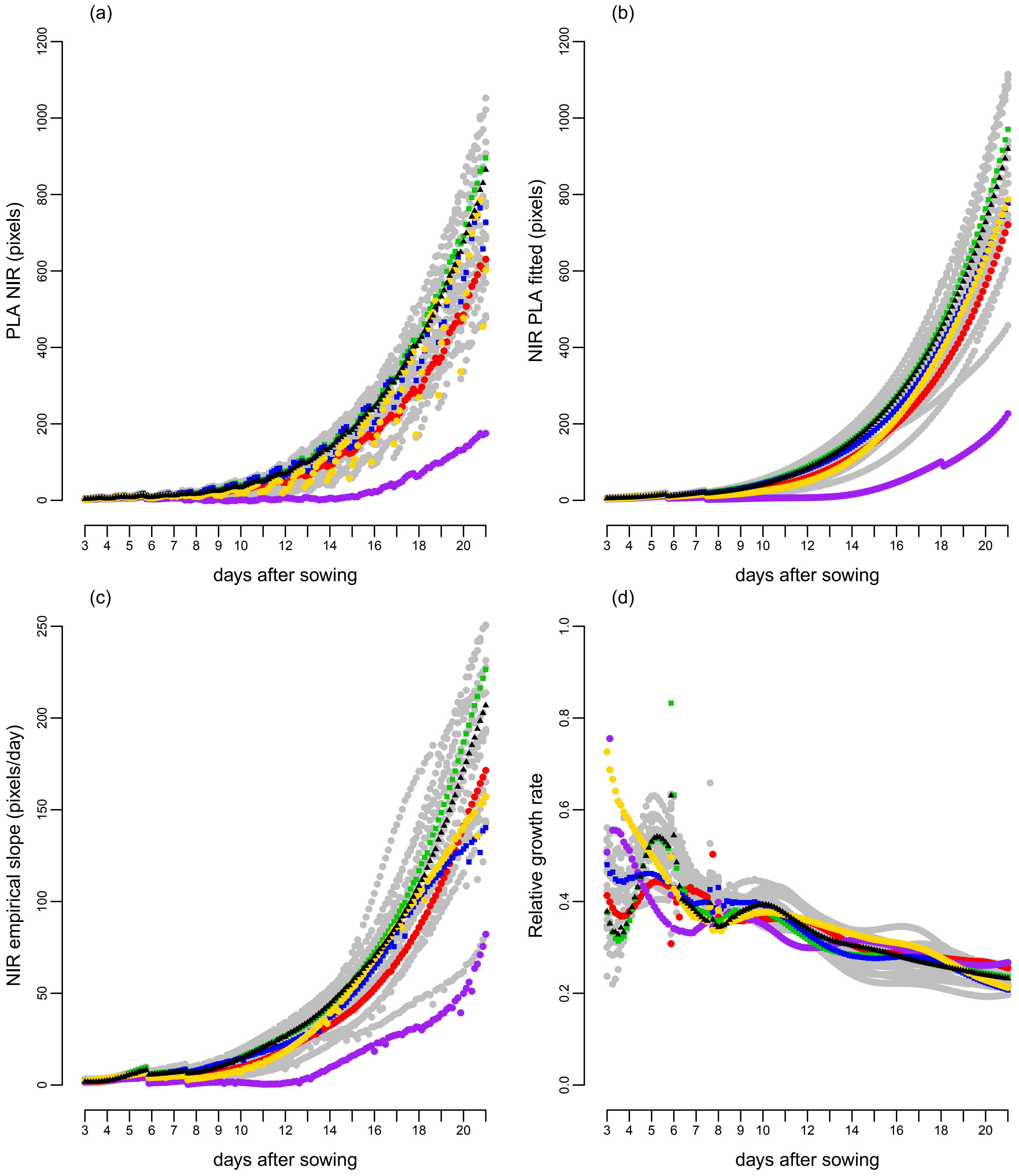

Figure S3. Phenotypic variation in growth curve parameters.

Phenotypic diversity in twenty Arabidopsis genotypes grown at 200 µmol m-2 s-1 light intensity. (a) Projected leaf area (PLA) from near infra-red (NIR) measurements. (b) Data from (a) fitted to a curve. (c) The empirical slope of the growth curve and (d) the relative growth rate. All data points are genotypic means (BLUEs), combining observations on replicates from different experiments into one representative value for each genotype at each time point. Six genotypes, An-2 (yellow circles), BC354 (purple circles), Bur-0 (green squares), Col-0 (black triangles), Ely (red circles) and Ts-1 (blue squares) are indicated in colour. Error bars have been excluded for clarity, the significance of between genotype differences is apparent from the heritability estimates in Figure S5.
